# Predictors of anxiety in Parkinson’s disease: results from a 3-year longitudinal cohort study

**DOI:** 10.1007/s10072-022-06427-8

**Published:** 2022-10-11

**Authors:** Jessie S. Gibson, Joseph L. Flanigan, James T. Patrie, W. Alex Dalrymple, Madaline B. Harrison

**Affiliations:** 1 School of Nursing, University of Virginia, Charlottesville, VA USA; 2 Department of Neurology, University of Virginia, Charlottesville, VA USA; 3 Department of Public Health Sciences, University of Virginia, Charlottesville, VA USA

**Keywords:** Parkinson’s disease, Anxiety, REM sleep behavior disorder, Autonomic dysfunction

## Abstract

**Introduction:**

Anxiety symptoms are the most common neuropsychiatric manifestation of Parkinson’s disease (PD), contributing to decreased quality of life. Few longitudinal studies in PD samples have examined correlates of anxiety symptoms over time. Understanding predictor variables may help to identify novel targets for reducing anxiety in PD. The aim of this study was to identify predictors of anxiety symptoms over 3 years in a clinic-based PD cohort.

**Methods:**

Our cohort included patients with PD at an academic medical center in the Southeastern United States (*n* = 105). Visits included assessment of motor, psychiatric, and cognitive features, including neuropsychological testing. For our multivariate model, we selected 11 predictor variables with the most existing evidence or theoretical support for an association with anxiety symptoms in PD. Multivariate linear mixed model regression was performed to determine which variables were significantly associated with anxiety symptoms over time.

**Results:**

Over half of participants (57%) met the screening threshold for an anxiety disorder at some point during the study. Independent predictors of anxiety symptoms over time included symptoms of REM sleep behavior disorder (RBD) and dysautonomia.

**Discussion:**

In this PD sample, RBD and dysautonomia symptoms were significantly associated with anxiety symptoms over time. Each of these relationships has been reported in one of two prior longitudinal studies. Unlike prior studies, cognitive impairment was not a significant predictor of anxiety symptoms in our sample. Future research should confirm the direction and mechanisms underlying these relationships, including the potential for anxiety symptom reduction through treatment for RBD and dysautonomia.

## Introduction

Anxiety is a common non-motor manifestation of Parkinson’s disease (PD). Over one-fourth of PD patients report clinically significant anxiety symptoms, and anxiety disorders are common in PD, with an average point prevalence of 31% and lifetime prevalence up to 49% [1, 2]. Anxiety in PD patients has been associated with several negative outcomes including worse emotional well-being, daily functioning, and health-related quality of life, as well as perceived stigma and increased caregiver burden [3–5]. Traditional pharmacological management of anxiety in PD is complicated by the increased susceptibility of people with PD to side effects (e.g., anticholinergic or antidopaminergic effects) common with psychotropic medications, and alternative strategies for anxiety reduction are needed [6].

Proposed factors contributing to anxiety in PD are varied. It has been postulated that anxiety in PD patients is related to structural and functional changes associated with PD: e.g., loss of striatal dopamine receptors and abnormal connectivity of frontostriatal networks [7, 8]. Based on a review of the literature, Sagna and colleagues proposed a conceptual model that identified various psychosocial and disease-related factors contributing to the development of depression and anxiety disorders in PD [9]. Among these were deficient social support, stress, “motor complications,” and longer disease and PD treatment duration. Furthermore, that model identified negative outcomes of these mood disorders in PD patients including social and relational difficulties, motor symptom fluctuation, sleep impairment, and decreased activity involvement, life expectancy, and quality of life [9]. Importantly, though anxiety and depressive symptoms often co-occur in PD, they are distinct syndromes. With anxiety being more prevalent than depression in PD, it is important to distinguish unique predictors and outcomes of anxiety symptoms in PD patients [3].

Various predictors of anxiety in PD have been proposed; however, most studies on this subject have been cross-sectional. To our knowledge, only two prior studies have incorporated analyses of longitudinal data to ascertain predictors of anxiety in PD [10, 11]. Rutten et al. (2017) analyzed data from the Parkinson’s Progression Markers Initiative (PPMI), including data from 306 participants who were assessed annually over 2 years [11, 12]. The authors found that older age, probable REM sleep behavior disorder (RBD), and worse cognition at baseline were associated with more anxiety over time [11]. Zhu et al. followed a large sample (*n* = 409) of PD patients in the Netherlands, who were assessed annually over 5 years [10]. These investigators found that female gender, cognitive impairment, insomnia, daytime sleepiness, dysautonomia, and depressive symptoms predicted future anxiety symptoms [10]. However, Zhu and colleagues used the Hospital Anxiety and Depression (HADS) Anxiety subscale as their primary outcome measure, an instrument that lacks specificity for detecting anxiety in PD [13, 14]. Considering the common co-occurrence of anxiety and depressive symptoms in PD, anxiety instrument specificity is particularly important [3, 10]. Furthermore, Zhu et al. did not include a measure of RBD as a potential predictor variable in their model; thus, they were unable to test the conclusions of Rutten et al. regarding RBD as a significant anxiety correlate [10, 11].

Understanding predictors of anxiety symptoms in PD may help us to identify patients at risk for anxiety disorders. Interventions targeting modifiable predictors of anxiety could reduce anxiety symptom burden over time. These insights could facilitate earlier intervention, leading to better patient and caregiver outcomes in this highly anxious population. In order to expand the understanding of anxiety in PD and address some of the limitations of prior research (i.e., shorter follow-up length, lack of anxiety scale specificity, exclusion of previously reported predictor variables like RBD), we aimed to determine prevalence and predictors of clinically significant anxiety symptoms in a PD cohort over 3 years.

## Methods

### Participants

We recruited patients at University of Virginia’s outpatient Movement Disorders Clinic to a 3-year observational study investigating psychiatric symptoms of PD. The University of Virginia Institutional Review Board approved the study, and all participants provided written informed consent. We enrolled participants beginning in March 2013, and assessed participants in follow-up through September 2019.

Patients with PD but without a clinical diagnosis of dementia were referred to the study team by their movement disorders neurologists. PD diagnosis was confirmed at screening by a movement disorders neurologist. All participants were required to have bradykinesia and at least one of the three other cardinal features of PD: resting tremor, rigidity, or postural instability. Patients were excluded if they were participating in an experimental trial to treat PD at any point during the study, though this had minimal effects on recruitment. Additionally, in order to remove patients with undiagnosed dementia, participants with a Montreal Cognitive Assessment (MoCA) score indicative of dementia (MoCA < 21) at baseline were excluded [15, 16]. Patients with dementia were excluded due to concerns that severe cognitive impairment would preclude accurate symptom self-reporting.

### Clinical assessment

Participants completed a baseline visit and up to three follow-up visits. Follow-up visits took place at intervals of approximately 1 year. Clinical and demographic information, as well as details regarding the onset of PD, were collected at baseline. At each visit, participants were administered the Movement Disorder Society Unified Parkinson Disease Rating Scale (MDS-UPDRS) [17], the Scale for the Assessment of Positive Symptoms [18], a structured interview regarding symptoms of psychosis, the Brief Psychiatric Rating Scale [19], the Apathy Scale [20], and a battery of neuropsychological assessments including the MoCA. The MDS-UPDRS was completed by a movement disorders neurologist. Additionally, at each time point, participants reported their current anti-Parkinsonian medications and dosages, and completed the following questionnaires: Beck Anxiety Inventory (BAI) [21], REM Sleep Behavior Disorder Screening Questionnaire (RBDSQ) [22], Scales for Outcomes in Parkinson’s-Autonomic Dysfunction (SCOPA-AUT) [23], and Beck Depression Inventory-II (BDI-II) [24], as well as additional clinical variables that were not included in the current analyses.

For this analysis, clinically significant anxiety symptoms, measured by BAI, were the primary outcome of interest. The BAI scores for the analyzable cohort of PD subjects ranged from 0 to 57, with higher scores indicating a greater level of anxiety. A score of 12 or higher on the BAI was previously observed to be an optimal cutoff score to indicate the presence of an anxiety disorder in PD [13]. BAI has moderate to good specificity for detecting anxiety in PD [13, 14].

### Statistical analysis

#### Data summarization

Categorical variables are summarized by frequencies (*n*) and relative frequencies (%). Ordinal scaled data (e.g., scores) and continuous scaled data (e.g., age) are summarized either by the mean and standard deviation of the empirical distribution, or by the median and interquartile range (IQR) of the empirical distribution.

#### Anxiety scale transformation

For the statistical analyses, the BAI scores were rescaled to the natural logarithmic scale, and since there were BAI scores equal to 0, the value of 1 was added to each BAI score before the natural logarithmic transformation was applied to the BAI score. This scale transformation was undertaken so that the anxiety data were more aligned with the analytical model assumptions.

#### Anxiety predictor variables

A set of predictor variables, previously reported in the literature as being associated with anxiety in PD, were examined as predictors of anxiety in this cohort of PD patients. Based on our sample size (*n* = 105), we determined that we could include 11 predictor variables in our multivariate model. We identified variables previously assessed in association with anxiety in PD then selected the variables that were most frequently found to relate to anxiety or had a strong theoretical basis for influencing PD anxiety symptoms. The predictor variables are as follows (references for each variable denote prior studies reporting a significant correlation and/or theoretical relevance): sex [10, 25–27], age at visit [11, 26, 28], age at onset of PD symptoms [26, 29], levodopa equivalent daily dosage (LEDD) [26, 30, 31], antidepressant use (yes/no) [2], dysautonomia symptoms (measured using SCOPA-AUT total score) [10, 25, 32], RBD symptoms (RBDSQ total score) [11, 25], cognitive impairment (MoCA total score) [10, 11], motor symptom severity (MDS-UPDRS Part III total score) [26, 28], PD subtype [29, 33], and complications of therapy [27–29, 34]. We assigned PD subtype (tremor dominant (TD), postural instability, and gait dysfunction (PIGD), or indeterminate (IND)) using the MDS-UPDRS in accordance with Stebbins et al. [35]. A binary categorical variable indicated the presence or absence of complications of therapy. The presence of complications of therapy was defined as a score greater than 0 on question 4.1 (dyskinesias) or 4.3 (fluctuations) of the MDS-UPDRS Part IV.

#### Univariate linear mixed model regression analyses

Univariate linear mixed model regression (LMMR) analyses were conducted to ascertain per predictor variable if the predictor variable was linearly associated with level of anxiety, i.e., the rescaled BAI score. Hypothesis testing was focused on determining if the value(s) of the LMMR coefficient(s) associated with the predictor variable is equal to zero, i.e., under the null hypothesis, it is assumed that there is no linear association between the predictor variable and the level of anxiety, i.e., the rescaled BAI score. The decision rule to reject the null hypothesis was based on the value of the *F*-statistic associated with LMMR predictor variable. An F-statistic exceeding the 95th percentile of null F distribution was established a priori as the level of confirmatory evidence needed to reject the null hypothesis.

#### Multivariate mixed model regression analysis

A multivariate LMMR analysis was conducted to ascertain if any of the 11 predictor variables is uniquely linearly associated with level of anxiety (i.e., the rescaled BAI score) after accounting for the variability in the level of anxiety that is explained by the remaining predictor variables. Hypothesis testing was focused on testing if the value(s) of the LMMR coefficient(s) associated with the predictor variable is equal to zero, i.e., under the null hypothesis, it is assumed that there is no unique linear association between level of anxiety and the predictor of level of anxiety after accounting for the variability in the level of anxiety that is explained by the remaining predictor variables. The decision rule to reject the null hypothesis was based on the value of the type III *F*-statistic associated with LMMR predictor variable. An F-statistic exceeding the 95th percentile of null *F* distribution was established a priori as the level of confirmatory evidence needed to reject the null hypothesis.

## Results

### Patient sample

Of the 116 participants recruited to the study, 11 participants scored less than 21 on the MoCA at baseline and were excluded from the present investigation. The remaining 105 participants met eligibility criteria and completed baseline visits. Of these participants, 92 completed the year 1 visit, 74 completed the year 2 visit, and 67 completed the year 3 visit. Reasons for attrition in enrollment include early participant withdrawal (*n* = 21), loss of contact with participant (*n* = 4), death of participant (*n* = 3), and study closure prior to the completion of the participant’s follow-up period (*n* = 10). Participants had a median total follow-up time of 3 years (IQR: [1.9, 3.0]) and a median time between annual evaluations of 12.2 months (IQR: [11.5, 13.1]).

Of the 338 visits completed by participants, three visits were missing BAI scores and were not included in univariate or multivariate analyses. Of the remaining 335 visits, the following predictor data were missing: 9 RBDSQ scores, 2 MoCA scores, 2 SCOPA-AUT scores, and 10 PD-subtype classifications. No data were missing for age, age of onset, LEDD, MDS-UPDRS Part III score, or the indicator for complications of therapy.

### Clinical characteristics

The baseline characteristics of the sample are summarized in Table 1. The sample was primarily male, with a median disease duration of less than 5 years. Three participants were not on anti-Parkinsonian medication at baseline. Mean baseline MoCA score (24.8) indicated mildly impaired cognition. The median BAI score was below the screening cutoff for the presence of an anxiety disorder at baseline, but the mean score increased at each visit (see Table 2). Applying the previously established cutoff to our data, 40 participants (38%) met screening criteria for an anxiety disorder at baseline, and 60 participants (57%) met criteria for at least one visit during the study. There are no established cutoffs for the presence of depressive disorders in PD using BDI-II, but compared to general population cutoffs and previously reported averages in PD patients with depressive disorders, this median score in our sample indicates a relatively low burden of depression [24, 36]. It is unclear whether this was related to actual lack of depressive symptoms or to adequate treatment. Forty-five participants (43%) reported taking an antidepressant for at least one visit during the study. Three participants who were taking an antidepressant at baseline stopped it at some point during the study; seven participants who were not taking an antidepressant at baseline were taking an antidepressant at least one follow-up visit. Six participants (6%) underwent neurosurgical procedures to treat PD during the study.

**Table 1:** Participant baseline characteristics (*N* = 105)

Males, *n* (%)	62 (59.0)
Age, y, *μ* (SD)	67.8 (8.0)
Education, y, *μ* (SD)	16.3 (3.0)
Age at symptom onset, y, *μ* (SD)	61.9 (8.9)
Duration of disease from onset, *y*, median [IQR]	4.9 [3.4–7.7]
Total MDS-UPDRS Part III score, median [IQR]	24 [16–36]
PD subtype, *n* (%), (*n* = 101)	
Tremor dominant	58 (57.4)
Postural instability/gait difficulty	35 (34.7)
Indeterminate	8 (7.9)
Complications of therapy, *n* (%)	58 (55.2)
Dyskinesias, *n* (%)	36 (34.3)
Fluctuations, *n* (%)	50 (47.6)
LEDD total, median [IQR]	525 [400–750]
MoCA score, *μ* (SD)	24.8 (2.3)
SCOPA-AUT total score, (*n* = 104), median [IQR]	13 [9–18]
RBDSQ total score, (*n* = 98), median [IQR]	5 [2–8]
BAI total score, median [IQR]	9 [5–16]

^*^n = 105 unless otherwise indicated. *MDS-UPRDS*, Movement Disorder Society Unified Parkinson Disease Rating Scale; *LEDD*, levodopa equivalent daily dosage; *MoCA*, Montreal Cognitive Assessment; *PD*, Parkinson’s disease; *RBDSQ*, REM Sleep Behavior Disorder Screening Questionnaire; *SCOPA-AUT*, Scales for Outcomes in Parkinson’s-Autonomic; *BAI*, Beck Anxiety Inventory

**Table 2:** Summarization of anxiety over 3 years

	B (*n* = 105)		Y1 (*n* = 90)		Y2 (*n* = 73)		Y3 (*n* = 67)	
	Mean (SD)	Range	Mean (SD)	Range	Mean (SD)	Range	Mean (SD)	Range
BAI score	10.82 (8.13)	0–50	10.89 (9.47)	0–55	11.18 (9.30)	0–55	13.64 (11.48)	0–57
Anxiety disorder*, *n* (%)	40 (38.10%)		33 (36.67%)		32 (43.84%)		30 (44.78%)	

^*^Defined as a BAI score of ≥ 12

### Predictors of anxiety

In univariate analyses, increases in LEDD and in scores on the MDS-UPDRS Part III, RBDSQ, and SCOPA-AUT were significantly associated with increase in the rescaled predicted BAI score (see Table 3). PD subtype was also significantly associated with anxiety (*p* = 0.001, see Table 4). The mean rescaled BAI scores were higher in PIGD than IND, and higher in IND than TD; however, a significant difference was only observed between the scores for the TD and PIGD subtypes (*p* < 0.001). Antidepressant use was significantly associated with lower anxiety scores (*p* = 0.006, see Table 4). Increase in MoCA score, indicating better cognitive functioning, was associated with decrease in the rescaled BAI score, but the relationship was not statistically significant (*p* = 0.06).

**Table 3:** Univariate mixed model regression analyses for predicting anxiety

Predictor	Subjects*	Observations*	Intercept	Lower 95% CI	Upper 95% CI	Regression coefficient	Lower 95% CI	Upper 95% CI	*p* value**
Age at visit	105	335	1.3578	0.1555	2.5600	0.0127	− 0.0047	0.0301	0.150
Age of onset	105	335	1.9002	0.8816	2.9188	0.0053	− 0.0110	0.0216	0.518
LEDD	105	335	2.0223	1.7815	2.2631	0.0003	0.0000	0.0006	0.036
MDS UPDRS III	105	335	1.8902	1.6434	2.1370	0.0114	0.0046	0.0183	0.001
RBDSQ	105	326	1.8962	1.6942	2.0982	0.0693	0.0396	0.0991	< 0.001
MoCA	105	333	2.9465	2.1808	3.7122	− 0.0287	− 0.0587	0.0014	0.061
SCOPA-AUT	105	333	1.4492	1.2511	1.6472	0.0550	0.0436	0.0664	< 0.001

*P* < .05

^*^Subjects = the number of subjects contributing data to the model, Observations = the total number of visits at which the respective predictor and BAI score were non-missing

^**^Ho: regression coefficient = 0

**Table 4:** Univariate mixed model regression analyses for predicting anxiety

	Mean log(BAI + 1)	Lower 95% CI	Upper 95% CI	Global *p* value	Comparison	*p* value
Subtype*						
TD	2.06	1.89	2.23	0.001	TD vs IND	0.063
IND	2.27	2.04	2.49		IND vs PIGD	0.168
PIGD	2.42	2.24	2.60		TD vs PIGD	< 0.001
Complications**						
No	2.24	2.06	2.41	0.887		
Yes	2.23	2.07	2.38			
Sex*						
Female	2.22	1.99	2.44	0.861		
Male	2.24	2.05	2.43			
Antidepressant use***						
No	2.44	2.24	2.64	0.006		
Yes	2.12	1.96	2.28			

*P* < .05

*BAI*, Beck Anxiety Inventory; *CI*, confidence interval; *IND*, indeterminate Parkinson’s subtype; *PIGD*, postural instability/gait dysfunction Parkinson’s subtype; *TD*, tremor-dominant Parkinson’s subtype

^*^No. of subjects = 105, no. of observations = 335

^**^No. of subjects = 102, no. of observations = 325

^***^No. of subjects = 102, no. of observations = 324

In multivariate analysis, the SCOPA-AUT and RBDSQ scores were significant independent predictors of change in anxiety (see Table 5). A one-unit increase in the SCOPA-AUT score, indicating more autonomic symptoms, was associated with an increase of 0.043 units in the rescaled BAI score ($\widehat{\beta }$= 0.043, 95% CI: [0.03, 0.057]; *F*_1, 268.9_ = 40.99, *p* < 0.001). A one-unit increase in the RBDSQ score, indicating more symptoms of RBD, was associated with an increase of 0.05 units in the predicted value of the rescaled BAI score ($\widehat{\beta }$= 0.05, 95% CI: [0.021, 0.078]; *F*_1, 262.9_ = 11.71, *p* = 0.001). A one-unit increase in the MDS-UPDRS Part III score also increased the rescaled BAI score, but the relationship was not statistically significant ($\widehat{\beta }$= 0.007, 95% CI: [0.000, 0.0138], *p* = 0.055). Similarly, antidepressant use as a predictor of rescaled BAI score approached but did not reach statistical significance ($\widehat{\beta }$= 0.199, 95% CI: [− 0.002, 0.4], *p* = 0.052).

**Table 5:** Multivariate mixed model regression for predicting change in anxiety

	Regression parameter estimate	95% CI	Degrees of freedom	*F*-statistic	*p* value
Intercept*	1.9491	[0.6413, 3.2568]			
Sex	0.2130	[− 0.0359, 0.4620]	(1, 104.8)	2.88	0.093
Age	− 0.0218	[− 0.055, 0.0115]	(1, 159.9)	1.67	0.198
Onset Age	0.0155	[− 0.0145, 0.0456]	(1, 145.1)	1.05	0.308
Levodopa dosage	0	[− 0.0003, 0.0003]	(1, 229.6)	0.09	0.767
MDS UPDRS III Score	0.0068	[− 0.0002, 0.0138]	(1, 256.1)	3.71	0.055
MoCA Raw Score	− 0.0191	[− 0.0476, 0.0093]	(1, 273.6)	1.75	0.187
RBDSQ Score	0.0495	[0.0210, 0.0780]	(1, 262.9)	11.71	0.001
SCOPA AUT Score	0.0432	[0.0299, 0.0565]	(1, 268.9)	40.99	< 0.001
Complications = Yes	− 0.0407	[− 0.1981, 0.1167]	(1, 284.5)	0.26	0.611
Subtype = IND	0.0644	[− 0.1414, 0.2702]	(2, 282.8)	0.89	0.411
Subtype = PIGD	0.1260	[− 0.0598, 0.3119]			
Antidepressant use = yes	0.1992	[− 0.0015, 0.3999]	(1, 204.7)	3.83	0.052
Model			(12, 197.0)	9.09	< 0.001

No. of subjects = 102; no. of observations = 312. *P* < .05

^*^Sex = F, Complications = no, Subtype = TD, Antidepressant use = no)

## Discussion

In a sample of PD patients, we found that symptoms of RBD and autonomic dysfunction were predictor variables associated with anxiety symptoms over 3 years. Each of these variables has been significantly associated with anxiety symptoms in one of the two previous longitudinal studies of anxiety predictors in PD [10, 11].

Rutten et al. reported that presence of probable RBD at baseline was associated with increases in anxiety scores (State-Trait Anxiety Inventory total and trait anxiety scores) over time [11]. Although Zhu et al. did not assess symptoms of RBD in their study, they did report that nighttime sleep problems were significantly associated with anxiety symptoms over time in their multivariate model [10]. Given that sleep issues are known to be worse in PD patients with RBD [37], it is possible that the relationship between sleep problems and anxiety in Zhu et al.’s sample could have been mediated by RBD symptoms. Considering the present findings, the relationship between RBD and anxiety symptoms has been consistently demonstrated across samples, using various measures of anxiety, and supports what has been reported in cross-sectional studies [25, 38]. In future studies, this finding could be further supported with diagnostic confirmation of RBD through polysomnography. In practice, clinicians may consider prioritizing anxiety screening for newly diagnosed PD patients with RBD. Furthermore, because RBD may worsen as PD advances, this is one more reason that screening for and treating anxiety should remain central to PD care throughout the disease course [39]. Finally, considering this relationship, treatment for RBD may help to reduce anxiety symptoms in people with PD. Melatonin supplementation is a common RBD treatment that was shown to decrease PD anxiety symptoms in a recent RCT [40]. In future studies, researchers should continue to investigate the potential for anxiety reduction via treatment for RBD.

Evidence to support the dysautonomia-anxiety relationship over time in PD is less established. Similar to our results, Zhu et al. found that dysautonomia scores (specifically SCOPA-AUT gastrointestinal and cardiovascular subscales) were associated with higher anxiety scores in their PD sample [10]. On the other hand, Rutten et al. did not find SCOPA-AUT scores to be significantly associated with anxiety over time [11]. Our subjects, who had more advanced disease than PPMI subjects, had higher baseline SCOPA-AUT total scores (mean = 13.2) than Rutten et al.’s subjects (mean = 8.9). It may be that Rutten and colleagues were unable to detect a significant effect of autonomic dysfunction symptoms on anxiety symptoms due to their sample’s earlier disease stage and consequent lower autonomic symptom burden. The inconsistencies between study findings could also be related to the anxiety measures used, as some subjective symptoms of anxiety and dysautonomia can be difficult to distinguish. For example, the BAI includes items querying dizziness, indigestion, lightheadedness, and feelings of choking as anxiety symptoms, all of which can be manifestations of autonomic dysfunction and are similar to items on the SCOPA-AUT. Therefore, it is possible that these two measures are assessing the same symptoms, and it would be difficult to discern the true etiology of the symptoms without further investigation. The ill-defined distinction between some anxiety and dysautonomic symptoms is a well-known clinical phenomenon. Indeed, anxiety disorders can be characterized by features of autonomic dysfunction (e.g., decreased heart rate variability due to impaired vagal response, tachycardia), and treatment for autonomic dysregulation can improve self-reported anxiety symptoms [41, 42]. This is seen clinically in PD, where a patient may report that anxiety symptoms improve with treatment for orthostatic hypotension [43]. Longitudinal studies in this population, including more objective measures of autonomic function and anxiety symptoms (i.e., autonomic function testing), could better define the differences (and overlap) of these phenomena, further testing the idea that treatment for dysautonomia may improve anxiety in PD. Conversely, pharmacological and/or behavioral treatment for anxiety symptoms (e.g., cognitive behavioral therapy) could improve perceived dysautonomia symptoms, given the broad overlap in symptomatology.

Unlike the two prior studies, we did not find cognitive impairment to be associated with anxiety symptoms over time. This may be explained, in part, by differences in baseline characteristics between our sample and prior samples. At baseline, compared to Rutten et al.’s sample, our sample was older, more cognitively impaired, and not levodopa-naïve, with greater time since PD diagnosis, higher average motor scores, and a greater proportion of females. Compared to Zhu et al.’s sample, our sample was older, with an older age of disease onset, lower average disease duration, and greater proportion of females. Rates of cognitive decline vary based on age of PD onset (e.g., faster decline in late-onset PD, slower decline in early-onset PD) and patterns of cortical degeneration in PD vary based on disease duration [44, 45]. Thus, it is possible that the older age and later PD onset in our sample could indicate disease characteristics (i.e., faster symptom progression, greater cortical atrophy) that potentially explain the disparate findings between our sample and prior samples.

In our sample, motor scores were significantly associated with anxiety scores in univariate analyses and trended toward significance in multivariate analyses. Prior studies have reported similar results. Zhu et al. found that motor scores, measured with the Short Parkinson’s Evaluation Scale/SCOPA-motor impairment, were significantly higher in PD patients with anxiety at baseline compared to those without anxiety (*p* = 0.004) [10, 46]. This relationship has been shown in multiple cross-sectional samples [26, 28]. However, as in the current study, both Zhu et al. and Rutten et al. did not find motor impairment to be significantly associated with anxiety scores over time [10, 11]. Additionally, RBD and dysautonomia may identify patients with a more rapidly progressive malignant PD subtype, often associated with worse cognitive and motor function [47, 48]. In such a subpopulation, cognitive and motor scores may be less likely to be independent predictors. Thus, although motor and cognitive symptoms were not significant predictors of anxiety on multivariate analysis in this sample, future studies could consider including these variables, particularly to distinguish anxiety predictors among those with and without the diffuse malignant PD subtype.

Similar to motor symptom burden, antidepressant use was associated with lower anxiety scores on univariate analysis, but this relationship did not meet the threshold for significance in the multivariate model. This is consistent with the findings of Rutten et al., who reported that the addition of antidepressants and anxiolytics at follow-up had no confounding effects on their prediction models of anxiety scores [11]. Because we did not exclude participants who were taking anxiety-reducing medications (e.g., antidepressants or benzodiazepines) or participating in behavioral therapies, it is likely that anxiety symptoms were well-controlled in some of our participants. Only ten participants changed their antidepressant status (i.e., taking versus not taking an antidepressant) over the course of the study, further indicating that anxiety symptoms may have been well-treated in our sample. However, we did not measure anxiolytic or behavioral therapy use, which is a limitation, and we recommend inclusion of these variables in future multivariate models. Nonetheless, our results from this clinic-based sample reflect levels of symptom burden of treated patients in a real-world clinical setting. Our conclusions are generalizable to PD patients receiving similar levels of care for their neuropsychiatric symptoms, whether in a specialty neurology clinic or via a non-neurology clinician with PD and/or psychiatric expertise.

Over half of participants (57%) met criteria for an anxiety disorder at some point during the study, based on established BAI screening cutoffs [13]. This is somewhat higher than previously reported averages [1, 2]. Because this was a longitudinal study, we are likely to have captured more symptoms than typically seen in cross-sectional studies and in studies where estimates of lifetime anxiety prevalence are based on subject recall. However, BAI is primarily a screening tool. In fact, it was previously found that BAI has only fair sensitivity to determine the presence of anxiety syndromes in PD, and other instruments like the Parkinson Anxiety Scale are recommended to account for these limitations of the BAI in PD [13, 49]. However, considering prior work in this area (i.e., Zhu et al., 2017), BAI is more useful than previously used measures (i.e., HADS) for discriminating between anxiety and depressive symptoms in PD [13, 14] and is recommended for monitoring longitudinal change in anxiety symptom severity [50]. Thus, our results offer useful information regarding predictors of clinically significant anxiety symptoms in PD. In future studies, addition of diagnostic interviews or use of an alternative measure like the Parkinson Anxiety Scale could confirm prevalence of anxiety disorders over time and look for differences in predictors among anxiety subtypes.

As reported above, considering sample size limitations, we selected the 11 covariates for our multivariate model based on what had been previously reported about anxiety symptoms and associated variables in PD. It is possible that other excluded variables could have influenced our resulting model, and our sample size limited our ability to include additional variables in the analysis. However, our results are generally consistent with prior studies, barring some logical exceptions as noted above. While we acknowledge this as a limitation of our study, we do not expect that our overall findings would have been meaningfully different with the inclusion of additional covariates. To confirm this, future studies could include multiple sites, allowing for a larger sample and inclusion of additional covariates of interest.

On multivariate analysis, the following variables were not significantly associated with anxiety symptoms over time in this study and in one of the two previous longitudinal studies: age, sex, PD subtype, age of PD onset, motor complications (i.e., fluctuations and/or dyskinesia), antidepressant use, and LEDD [10, 11]. The variables discussed above (i.e., RBD and dysautonomia symptoms, cognitive impairment) have more evidence to support their potential significance in multivariate models of anxiety. This suggests that these three variables may have the most relevance for future studies to further define predictors of anxiety in PD. Although further study may be warranted to investigate the potential relevance of additional clinical variables as anxiety predictors (e.g., motor symptoms, antidepressant use), we do not recommend substituting these variables for those discussed above (i.e., RBD, dysautonomia, cognitive impairment).

This study has confirmed the importance of RBD symptoms as a predictor associated with anxiety symptoms over time in PD. The longitudinal influences of autonomic dysfunction, cognitive impairment should be re-examined in future studies. Replication of these findings and inclusion of diagnostic measures (i.e., polysomnography, autonomic function testing, psychiatric interview) would further support our hypothesis that RBD and dysautonomia could be targeted to facilitate anxiety reduction in PD. Future research should also aim to test relevant pharmacologic and non-pharmacologic methods (e.g., melatonin supplementation, biofeedback training, cognitive behavioral therapy) for reducing PD anxiety over time.
